# Crystal Structure of the Chloroplastic Oxoene Reductase ceQORH from *Arabidopsis thaliana*

**DOI:** 10.3389/fpls.2017.00329

**Published:** 2017-03-09

**Authors:** Sarah Mas y mas, Gilles Curien, Cécile Giustini, Norbert Rolland, Jean-Luc Ferrer, David Cobessi

**Affiliations:** ^1^Institut de Biologie Structurale (IBS), Univ. Grenoble Alpes, CEA, Centre National de la Recherche Scientifique (CNRS)Grenoble, France; ^2^Laboratoire de Physiologie Cellulaire & Végétale, BIG, Univ. Grenoble Alpes, CEA, Centre National de la Recherche Scientifique (CNRS), INRAGrenoble, France

**Keywords:** chloroplast envelope quinone oxidoreductase homolog, oxylipins, γ-ketols, α, β-unsaturated carbonyls, X-ray crystallography

## Abstract

Enzymatic and non-enzymatic peroxidation of polyunsaturated fatty acids give rise to accumulation of aldehydes, ketones, and α,β-unsaturated carbonyls of various lengths, known as oxylipins. Oxylipins with α,β-unsaturated carbonyls are reactive electrophile species and are toxic. Cells have evolved several mechanisms to scavenge reactive electrophile oxylipins and decrease their reactivity such as by coupling with glutathione, or by reduction using NAD(P)H-dependent reductases and dehydrogenases of various substrate specificities. Plant cell chloroplasts produce reactive electrophile oxylipins named γ-ketols downstream of enzymatic lipid peroxidation. The chloroplast envelope quinone oxidoreductase homolog (ceQORH) from *Arabidopsis thaliana* was previously shown to reduce the reactive double bond of γ-ketols. In marked difference with its cytosolic homolog alkenal reductase (AtAER) that displays a high activity toward the ketodiene 13-oxo-9(Z),11(E)-octadecadienoic acid (13-KODE) and the ketotriene 13-oxo-9(Z), 11(E), 15(Z)-octadecatrienoic acid (13-KOTE), ceQORH binds, but does not reduce, 13-KODE and 13-KOTE. Crystal structures of apo-ceQORH and ceQORH bound to 13-KOTE or to NADP^+^ and 13-KOTE have been solved showing a large ligand binding site, also observed in the structure of the cytosolic alkenal/one reductase. Positioning of the α,β-unsaturated carbonyl of 13-KOTE in ceQORH-NADP^+^-13-KOTE, far away from the NADP^+^ nicotinamide ring, provides a rational for the absence of activity with the ketodienes and ketotrienes. ceQORH is a monomeric enzyme in solution whereas other enzymes from the quinone oxidoreductase family are stable dimers and a structural explanation of this difference is proposed. A possible *in vivo* role of ketodienes and ketotrienes binding to ceQORH is also discussed.

## Introduction

**Figure 1:**
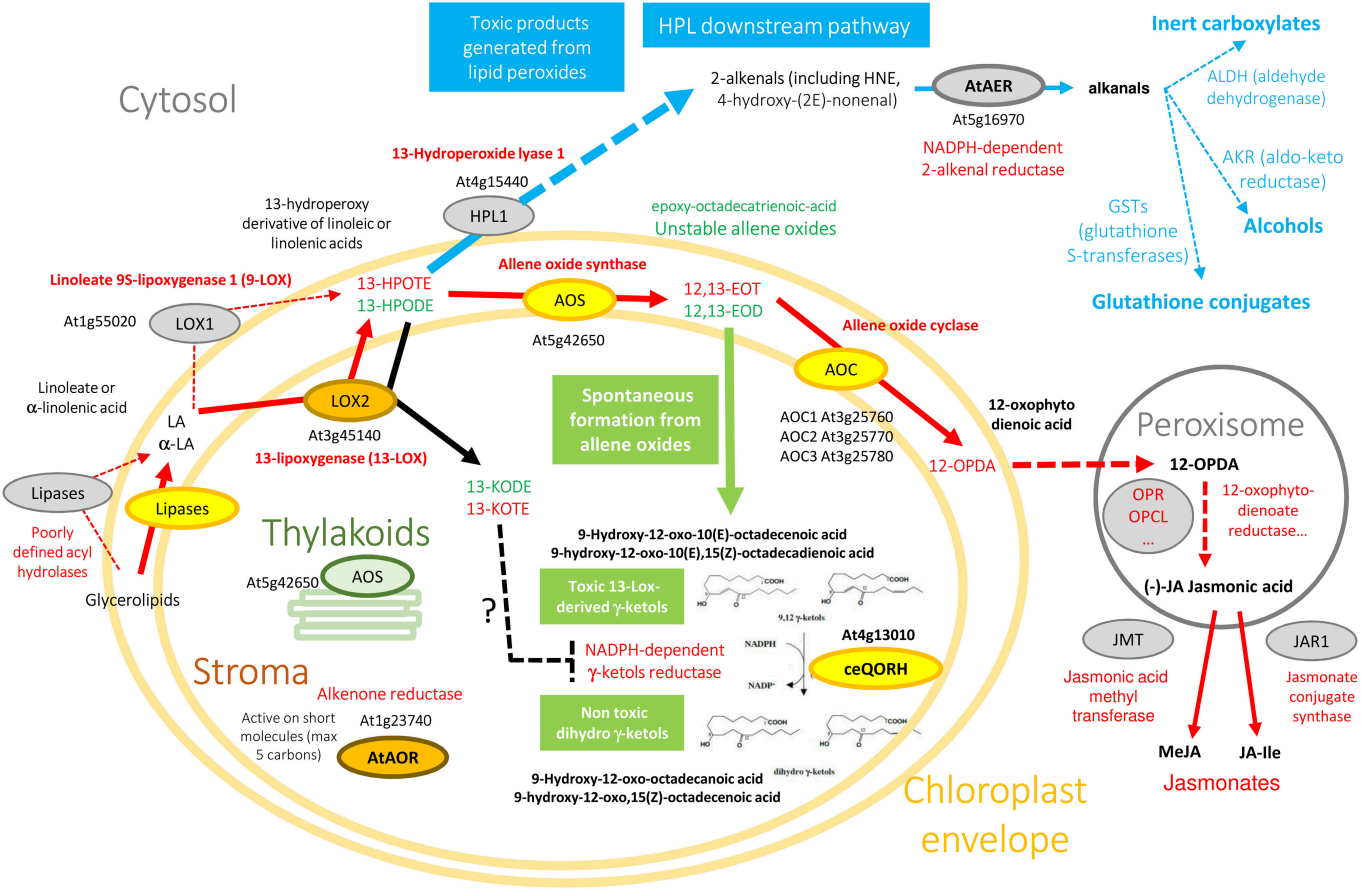
**Oxylipin metabolism engenders essential, but also toxic molecules**. Color code originates from experimental information about the subplastidial localization of enzymes (see AT_CHLORO database; Ferro et al., 2010). Yellow enzymes (AOS, AOC, ceQORH) are associated to the chloroplast envelope while brown ones (LOX2, AtAOR) are found in the chloroplast stroma. Note that AOS is associated with both envelope (yellow) and thylakoid membranes (green). Non-plastid enzymes (cytosol, peroxisomes), or proteins suspected to interact with the outer surface of chloroplasts, are colored in gray. Due to space constraints, some specific data are absent from this figure. For example, from the cytosolic 9-LOX (LOX1), derive 9-HPOTE and 9,10-EOT that are not indicated here, and α and γ-ketols also derive from 9,10-EOT. 13(*S*)-HPODE and 12,13-EOD derive from linoleic acid (18:2) while 13(*S*)-HPOTE and 12,13-EOT derive from linolenic acid (18:3). Note that from 13(*S*)-HPODE and 13(*S*)-HPOTE, LOX2 synthesize 13-KODE and 13-KOTE, respectively (black arrow).

Plants lack an immune system like animals. However, they possess mechanisms that recognize pathogens and initiate defense responses. Some of these mechanisms involve various types of oxygenated fatty acids, termed “oxylipins.” These molecules are involved in responses to physical damage by animals or insects, stress, and attack by pathogens. Oxylipins are derived from linoleic and α-linolenic acids, released from their lipid associations by poorly defined acyl hydrolases (lipases) of various kinds (Figure 1). A first key step in oxidation is the action of lipoxygenases. For example, depending on the source of the enzyme, lipoxygenases (e.g., LOX1, a 9-lipoxygenase from the cytosol, or LOX2, a 13-lipoxygenase from the chloroplast stroma) catalyze the oxidation of linoleic (C18:2) or linolenic acids (C18:3) into either 9- or 13-hydroperoxy-octadecatrienoic acids (HPODE). Such compounds are highly reactive, and they are quickly metabolized by various enzymes into series of oxylipins, with a range of distinct biological activities (Blée, 2002; Mosblech et al., 2009; Joyard et al., 2010). Some oxylipins such as 4-oxononenal, 4-hydroxynonenal, ketodienes [the 9-oxo-10(E), 12(E)-octadecadienoic acid (9-KODE), and 13-oxo-9(Z), 11(E)-octadecadienoic acid (13-KODE)], ketotrienes [the 9-oxo-10(E), 12(Z), 15(Z)-octadecatrienoic acid (9-KOTE) and 13-oxo-9(Z), 11(E), 15(Z)-octadecatrienoic acid (13-KOTE)], or the plant specific γ-ketols (Figure 1 and Figure S1) are α,β-unsaturated carbonyls and reactive electrophile species (RES). RES can act as signaling molecules (Farmer and Mueller, 2013) and also react with important cellular nucleophiles such as thiols of proteins and nucleic acids, modifying their biochemical properties, and are potentially toxic (Esterbauer et al., 1991; Szweda et al., 1993). Due to this potential toxicity, cells have developed an armory of defense to decrease their activity. One class of enzymes involved in detoxification of reactive electrophile oxylipins are NADPH-oxidoreductases belonging to the zinc-independent medium-chain dehydrogenase/reductase (MDR) superfamily and more specifically to the quinone oxidoreductase (QOR) subfamily to which alkenal/one reductases belong (Porté et al., 2009). These enzymes can decrease the activity of reactive electrophile oxylipins by reducing the unsaturated carbon-carbon bond located in α,β of the carbonyl group to a single bond (Yamauchi et al., 2011) (Figure 1).

The chloroplast envelope Quinone Oxidoreductase Homolog (ceQORH; At4g13010; molecular weight 34,034 Da) from *Arabidopsis thaliana* is associated to the inner membrane of the chloroplast envelope (Figure 1) where it represents 1–2% of the crude envelope proteins (Miras et al., 2002). It is encoded by the nuclear genome and is targeted to the chloroplast by an alternative import pathway, without cleavage of its internal chloroplast targeting sequence (Miras et al., 2002, 2007). Despite its original annotation as a “QOR,” ceQORH is inactive on quinones but preferentially reduces γ-ketols in the presence of NADPH (Curien et al., 2016) (Figure 1 and Figure S1). γ-ketols (Figure 1 and Figure S1) are long-chain reactive electrophile oxylipins and are potentially toxic (Kuga et al., 1993). They are spontaneously produced in the jasmonate biosynthetic pathway, downstream of lipoxygenase specific peroxidation by hydrolysis of an allene oxide intermediate (Grechkin et al., 1991). ceQORH is also active though to a lesser extent on the highly toxic (Lin et al., 2005) C9 α,β-unsaturated carbonyl 4-oxononenal (Curien et al., 2016). ceQORH is thus probably dedicated to detoxification of γ-ketols which can be produced in plants under normal growth conditions (Theodoulou et al., 2005), and accumulate in damaged tissues (Buseman et al., 2006) or at a distance from bite zone when plants are attacked by caterpillars (Schulze et al., 2007). ceQORH bears similar characteristics and sequence homology with members of the MDR superfamily, which includes chloroplastic alkenone reductase [At1g23740, AtAOR (Yamauchi et al., 2012), Figure 1], Arabidopsis ζ-crystallin [alkenal reductase, AT5G16970, AtAER (Mano et al., 2005), Figure 1], yeast ζ-crystallin (Crosas et al., 2011), human eye ζ-crystallin (Porté et al., 2011), and *Escherichia coli* quinone reductase (Thorn et al., 1995) (Figure S2).

Unlike the chloroplastic AtAOR (Yamauchi et al., 2012) (Figure 1), which is active on short-chain α,β-unsaturated carbonyls (C < 5), ceQORH does not reduce short chain unsaturated carbonyls (C < 9). In addition, compared to the cytosolic broad specific alkenal/one reductase AtAER (Figure 1), ceQORH showed a restricted substrate specificity, being inactive on the ketodienes 9-KODE and 13-KODE, or on the ketotrienes 9-KOTE and 13-KOTE (Figure S1) and virtually inactive on 4-hydroxynonenal and traumatin [12-oxo-10(E) dodecenoate] (Curien et al., 2016). ceQORH is encoded by the nuclear genome and is targeted to the chloroplast by an alternative import pathway independent from the trimeric TOC159/75/34 complex, without cleavage of its internal chloroplast targeting sequence (Miras et al., 2002, 2007). This peculiarity, together with the ceQORH restricted substrate specificity, prompted us to carry out crystallographic studies of ceQORH. No crystal was obtained either in the presence of γ-ketols or NADPH but the protein crystallized in the absence of ligands as well as bound to 13-KODE and NADPH, 13-KOTE and NADP^+^ and 13-KOTE alone. Structure comparisons with AtAER (Youn et al., 2006) and the enone oxidoreductase from *Fragaria x ananassa* (Schiefner et al., 2013) provided insights into the molecular basis of substrate specificity.

## Materials and methods

### Tryptophan fluorescence anisotropy measurements

Tryptophan fluorescence anisotropy measurements of ceQORH were carried out in the same experimental conditions as *in vitro* kinetics (Curien et al., 2016). Assays were carried out with a MOS-450 spectrometer (BioLogic, Inc.) in a 150 μL quartz cuvette, under temperature control (25°C). Excitation and emission wavelengths were set at 280 and 350 nm respectively. Assay conditions were: 10 mM HEPES pH 7.5, 150 mM KCl. Protein concentration was 200 nM. Changes in fluorescence anisotropy were probed following addition of NADPH (200 μM), 13-KODE (50 μM), or γ-ketols (100 μM).

### Crystallization and data collection

Expression, purification, crystallization, and data collection of apo-ceQORH were described previously (Mas y mas et al., 2015). ceQORH-13-KOTE (1.45 mM 13-KOTE) and ceQORH-13-KOTE-NADP^+^ (1.45 mM NADP^+^, 1.45 mM 13-KOTE) at 5 mg/ml in 50 mM Tris-HCl, pH 7.5, 200 mM KCl, 2 mM DTT, 1 mM EDTA, 10% (v/v) glycerol were subjected to crystallization using the sitting-drop vapor-diffusion technique and high throughput crystallization facility at EMBL, Grenoble, at 4°C. Crystallization hits were optimized using Limbro plates, at 20°C. Crystals of ceQORH-13-KOTE-NADP^+^ were obtained in 0.2 M sodium chloride, 0.1 M Tris-HCl pH 8.5, 15.5% (w/v) PEG 4K. Crystals of ceQORH-13-KOTE were obtained in 0.2 M ammonium tartrate pH 7.2, 20% (w/v) PEG3350. Crystals of ceQORH-NADPH-13-KODE (1.45 mM NADPH, 1.45 mM 13-KODE) were also obtained in 0.2 M sodium chloride, 0.1 M Tris-HCl pH 7.5, 28% (w/v) PEG3500, but despite all our efforts, it was not possible to obtain diffraction good enough and the resulting structure was not used here. All the diffraction data were collected on FIP-BM30A (Roth et al., 2002) at the European Synchrotron Radiation Facility, Grenoble, France, at 100 K, using an ADSC 315r detector. The data (Table 1) were processed and scaled using XDS (Kabsch, 2010).

**Table 1:** **Statistics of data collection**.

	**CeQORH-13-KOTE**	**CeQORH-NADP^+^-13-KOTE**
Resolution range (Å)	40.21–2.78 (2.94–2.78)	49.58–2.81 (2.98–2.81)
Wavelength (Å)	0.9796	1.04
Space group	P1	P2_1_
Unit cell parameters (Å,°)	a = 82.87, b = 121.01, c = 122.94, α = 66.73, β = 79.10, γ = 79.99	a = 82.16, b = 128.60, c = 150.12, β = 97.76
Molecules in au	12	8
Number of total reflections	186,642 (27,138)	283,856 (45,069)
Unique reflections	102,756 (15,212)	75,277 (11,943)
Average multiplicity	1.8 (1.8)	3.8 (3.8)
Data completeness (%)	94.8 (87.0)	99.4 (98.2)
Rsym (%)	14.5 (75.4)	8.6 (62.6)
<I/σ(I)>	6.1 (1.1)	13.7 (2.2)
CC(1/2)	ND	99.7 (77.0)

*Rsym = ΣΣ|Ii-Im|/ΣΣIi, where Ii is the intensity of the measured reflection and Im is the mean intensity of this reflection. Values indicated in parentheses correspond to the statistics in the highest resolution shell*.

### Phasing and model refinement

Phasing was performed by molecular replacement using Phaser (McCoy et al., 2007) from CCP4 (CCP4, 1994). The structure of the QOR from *Coxiella burnetii* (PDB entry: 3TQH) (Franklin et al., 2015) was used as model and modified based on sequence alignment with ceQORH using CHAINSAW (Stein, 2008) from CCP4 to calculate the phases for the data of ceQORH-13-KOTE. The other structures were solved using the monomer of ceQORH-13-KOTE as search model for molecular replacement. All the model refinements were performed with non-crystallographic symmetry. The refinements and rebuilding were done using PHENIX (Adams et al., 2010) and COOT (Emsley et al., 2010) respectively. Water molecules were added in apo-ceQORH using PHENIX. Refinement statistics are summarized in Table 2. Structure of ceQORH-NADPH-13-KODE was not used for analyses since statistics of X-ray data were poor. It is roughly similar to that of ceQORH-NADP^+^-13-KOTE and 13-KODE is located at a similar position than 13-KOTE.

**Table 2:** **Refinement statistics**.

	**Apo-ceQORH**	**CeQORH-13-KOTE**	**CeQORH-NADP^+^-13-KOTE**
Resolution (Å)	42.26–2.34 (2.42–2.34)	40.21–2.78 (2.81–2.78)	49.58–2.81 (2.84–2.81)
R_cryst_ (σ_F_ = 0) (%)	18.14 (22.19)	20.03 (32.28)	18.82 (32.46)
R_free_ (σ_F_ = 0) (%)	25.05 (32.92)	25.75 (36.90)	22.47 (38.87)
Number of atoms	5,275	28,354	19,576
Water molecules	387	0	0
B average (Å^2^)	25.64	45.46	72.36
Rmsd bonds (Å)	0.011	0.011	0.008
Rmsd angle (°)	1.350	1.286	1.214
Ramachandran favored (%)	98.04	97.02	97.80
Ramachandran outliers (%)	1.06	1.05	0.42

*Values indicated in parentheses correspond to the statistics in the highest resolution shell*.

*R_cryst_ = Σ||Fobs|-|Fcalc||/Σ|Fobs|. Rfree (Brünger, 1992) is the same as Rcryst but calculated for 5% data omitted from the refinement*.

Atomic coordinates and X-ray data were deposited in the PDB with the accession numbers: apo-ceQORH (5A3V), ceQORH-13-KOTE (5A3J), and ceQORH-NADP^+^-13-KOTE (5A4D).

## Results

### Analysis of ceQORH oligomerization state

The oligomeric forms of ceQORH (i.e., dimers and tetramers, see later) observed in the different crystals raised the question of their physiological relevance. Indeed, we previously showed by analytical ultracentrifugation analyses (AUC) (Mas y mas et al., 2015) that ceQORH in the presence of NADPH is a monomer while apo-ceQORH displayed several oligomerization forms, i.e., monomeric, dimeric, and tetrameric. As NADPH is always present *in vivo*, ceQORH is probably a monomer at least in the absence of the other substrates. To see whether γ-ketols (substrates) or the ketodiene and ketotriene (ligands) affected the ceQORH oligomerization state, tryptophan fluorescence anisotropy measurements of ceQORH were carried out in the same experimental conditions as *in vitro* kinetics (Curien et al., 2016). No change in fluorescence anisotropy was observed upon addition of NADPH, NADP^+^, 13-KOD(T)E or substrates, either alone or in combination. The same results were obtained when the fluorescence anisotropy was measured with a *C*-terminal GFP-fusion form of ceQORH taking opportunity of the fluorescence of GFP. Since ceQORH-NADPH is a monomer (Mas y mas et al., 2015) even at high concentration and no change in fluorescence anisotropy is observed upon addition of ligands we can conclude that ceQORH is active with its substrates and binds ligands as a monomer. The oligomeric states observed in AUC and in crystals (see after) thus result from the use of high ceQORH concentrations (17.5 and 174.9 μM in AUC and 145.8 μM in crystallogenesis) and probably do not have any physiological significance. The oligomerization state is however described and discussed in the context of structure comparison with other QORs that form stable dimers.

### The ceQORH oligomers

**Figure 2:**
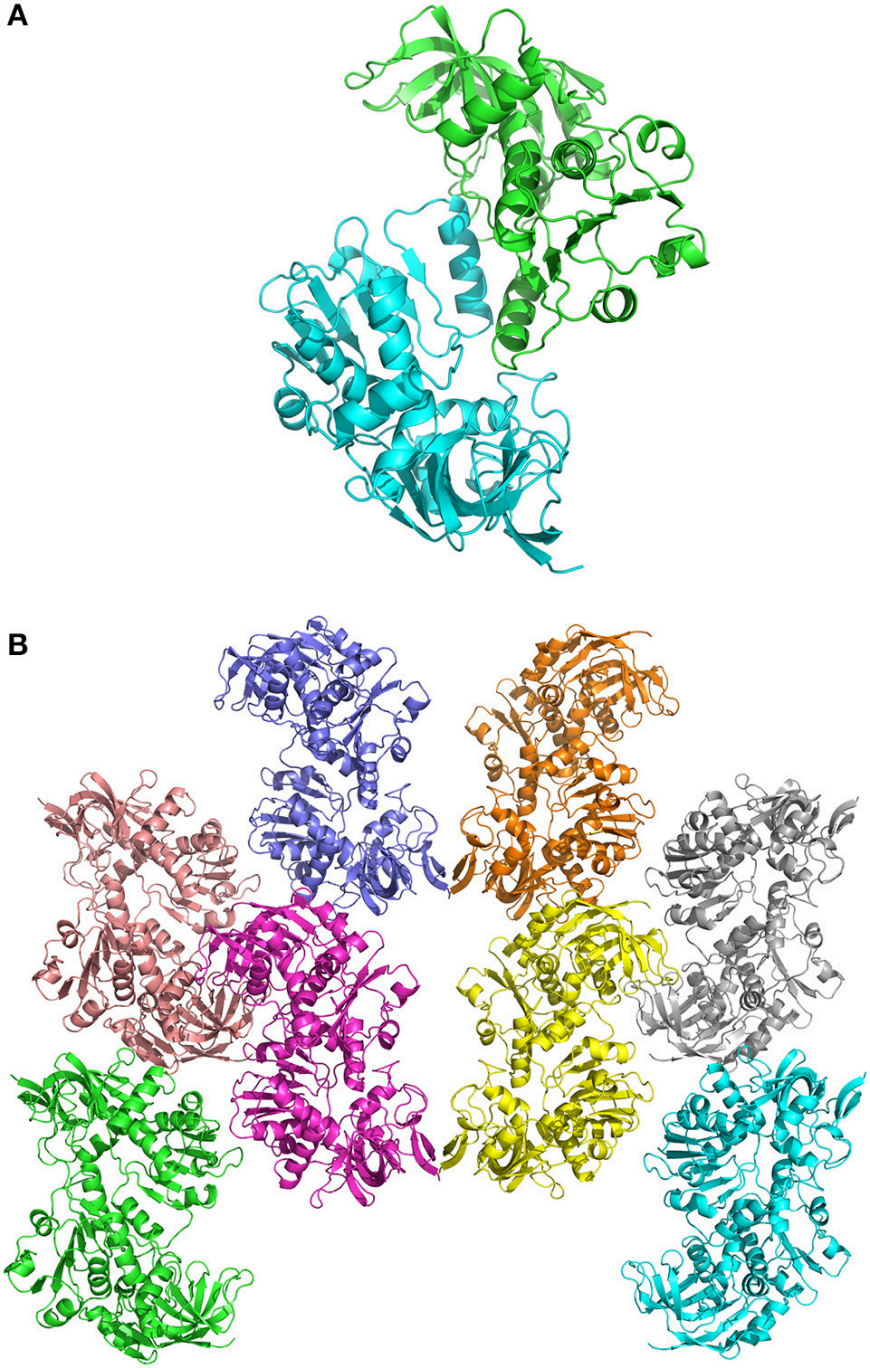
**(A)** view of the ceQORH dimer (apo-ceQORH) with each monomer in a different color. The β-strands are drawn in arrows and the α-helices are represented in ribbons. **(B)** crystal packing of the apo-ceQORH in the space group C222_1_. Two molecules are in the asymmetric unit.

**Figure 3:**
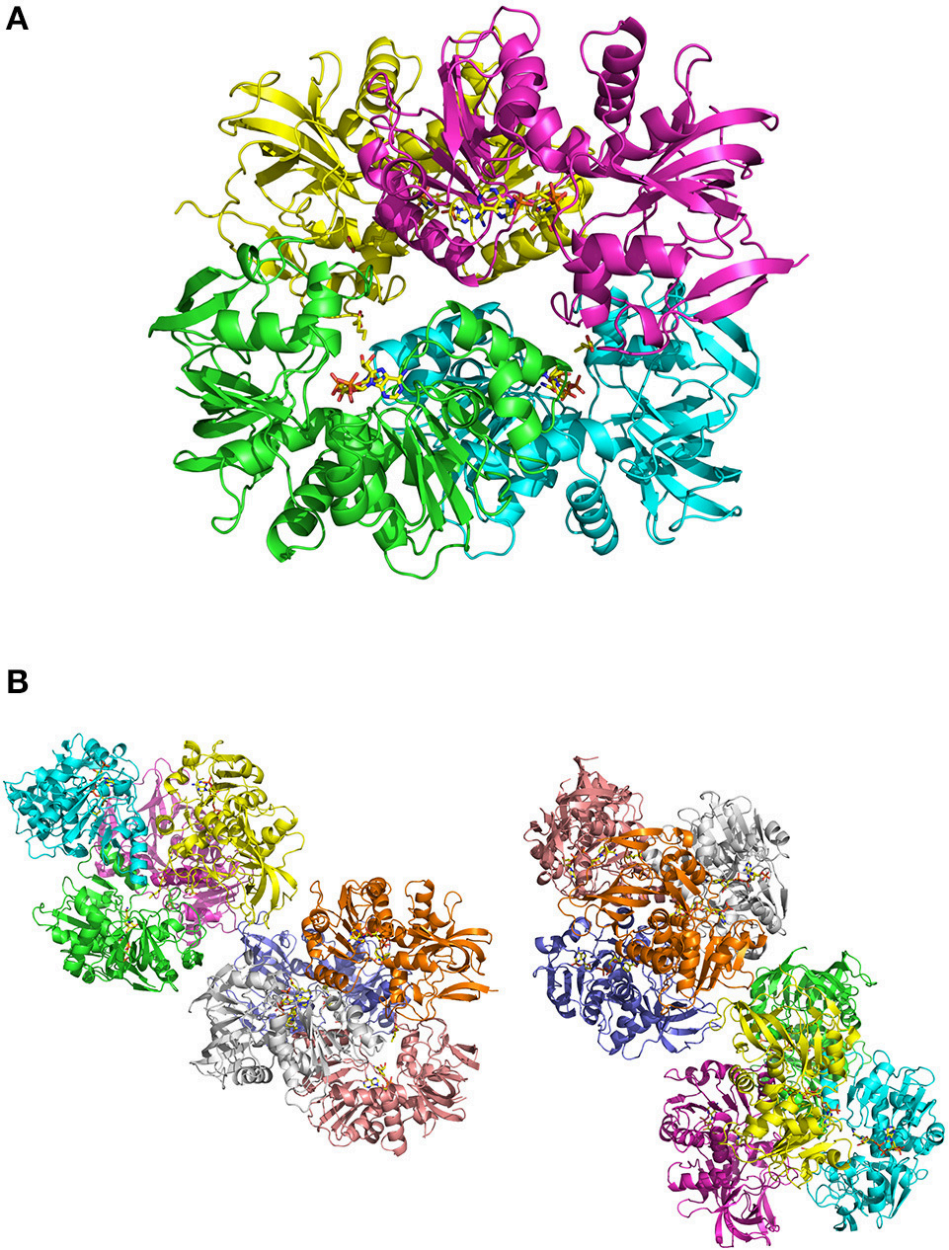
**(A)** view of ceQORH-NADP^+^-13-KOTE tetramer. Each monomer is displayed with a different color. The β-strands are drawn in arrows and the α-helices are represented in ribbons. NADP^+^ and 13-KOTE are drawn in sticks. **(B)** crystal packing of the ceQORH-NADP^+^-13-KOTE in the space group P2_1_. Eight molecules of ceQORH-NADP^+^-13-KOTE are in the asymmetric unit.

**Figure 4:**
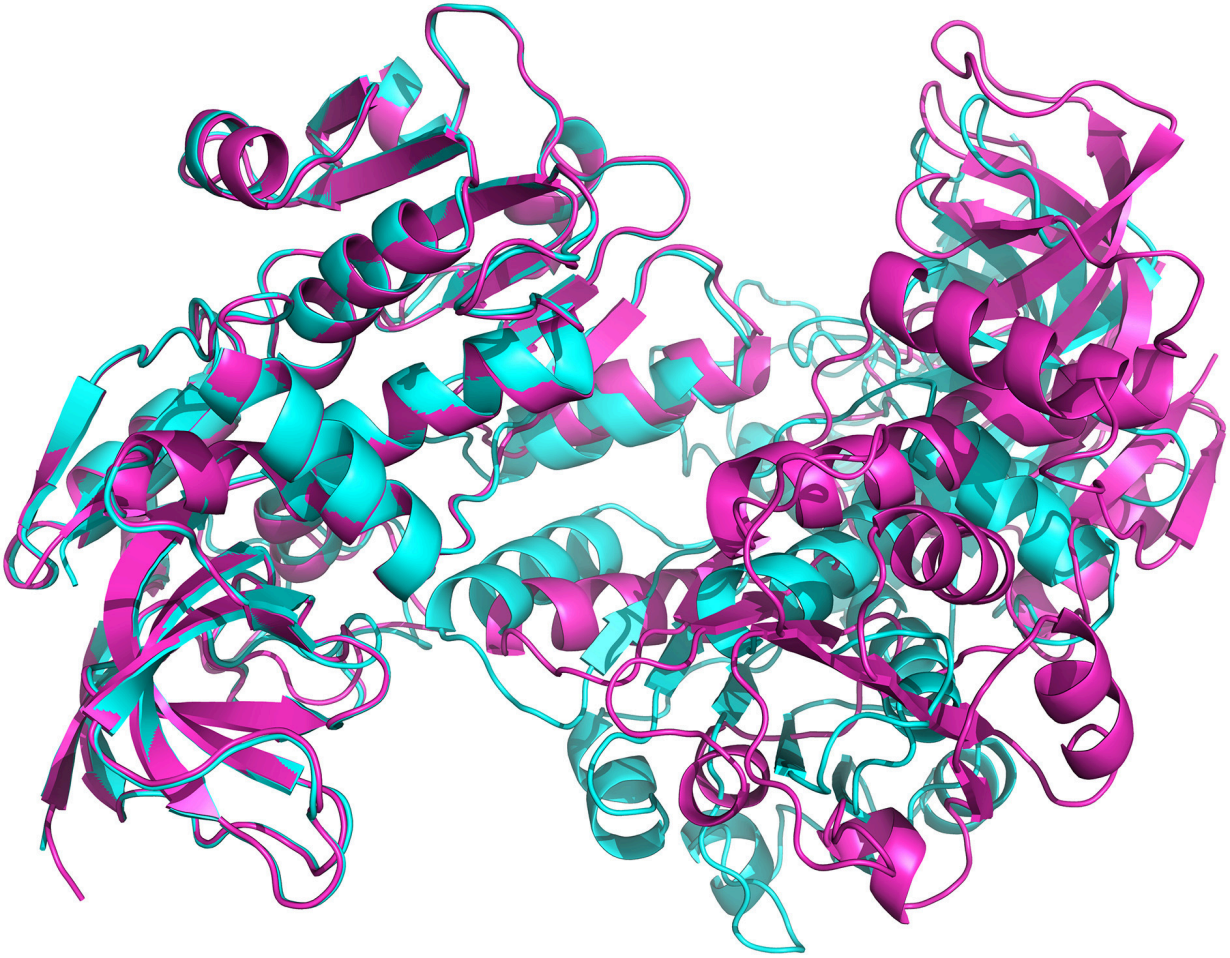
**View of the superimposition of apo-ceQORH (blue) onto ceQORH-NADP^+^-13-KOTE (purple)**. For clarity a single dimer of the tetramer was drawn.

In crystals of apo-ceQORH, the asymmetric unit contains two monomers related by a non-crystallographic two-fold axis (Figures 2A,B) that form a dimer. The monomers are very similar with a value of root mean square deviation (rmsd) of 0.25 Å between monomers. The buried area in the dimer interface is 2,410 Å^2^. The dimer of apo-ceQORH crystallized whereas monomers and tetramers observed in AUC did not. No crystallization could be observed with the following complexes, ceQORH-NADPH, ceQORH-γ-ketol, and ceQORH-NADP^+^-γ-ketols using either 18:1 or 18:2 γ-ketols. We could obtain crystals with 13-KODE and 13-KOTE without and with NADPH or NADP^+^. In the crystals of the binary and ternary complexes ceQORH-13-KOTE and ceQORH-NADP^+^-13-KOTE, the asymmetric unit contains 12 and 8 ceQORH molecules, respectively. They assemble into tetramers (Figures 3A,B). As indicated above the tetramers observed in the crystals do not have any physiological significance. The structure of the tetramers is similar as characterized by low values of rmsd when the tetramers are superimposed, in a range from 0.62 to 1.19 Å. The monomers of the three ceQORH structures can be superimposed with low rmsd going from 0.03 to 0.80 Å. The highest differences in rmsd are observed between the monomers of apo-ceQORH and those in complexes. The dimer of apo-ceQORH does not superimpose on any dimers of the tetramers (Figure 4). The interactions between monomers are therefore the main differences between the apo-ceQORH dimer and the tetramers.

### Comparison of apo-ceQORH and ceQORH bound to ligands

#### Overall description of the monomers

**Figure 5:**
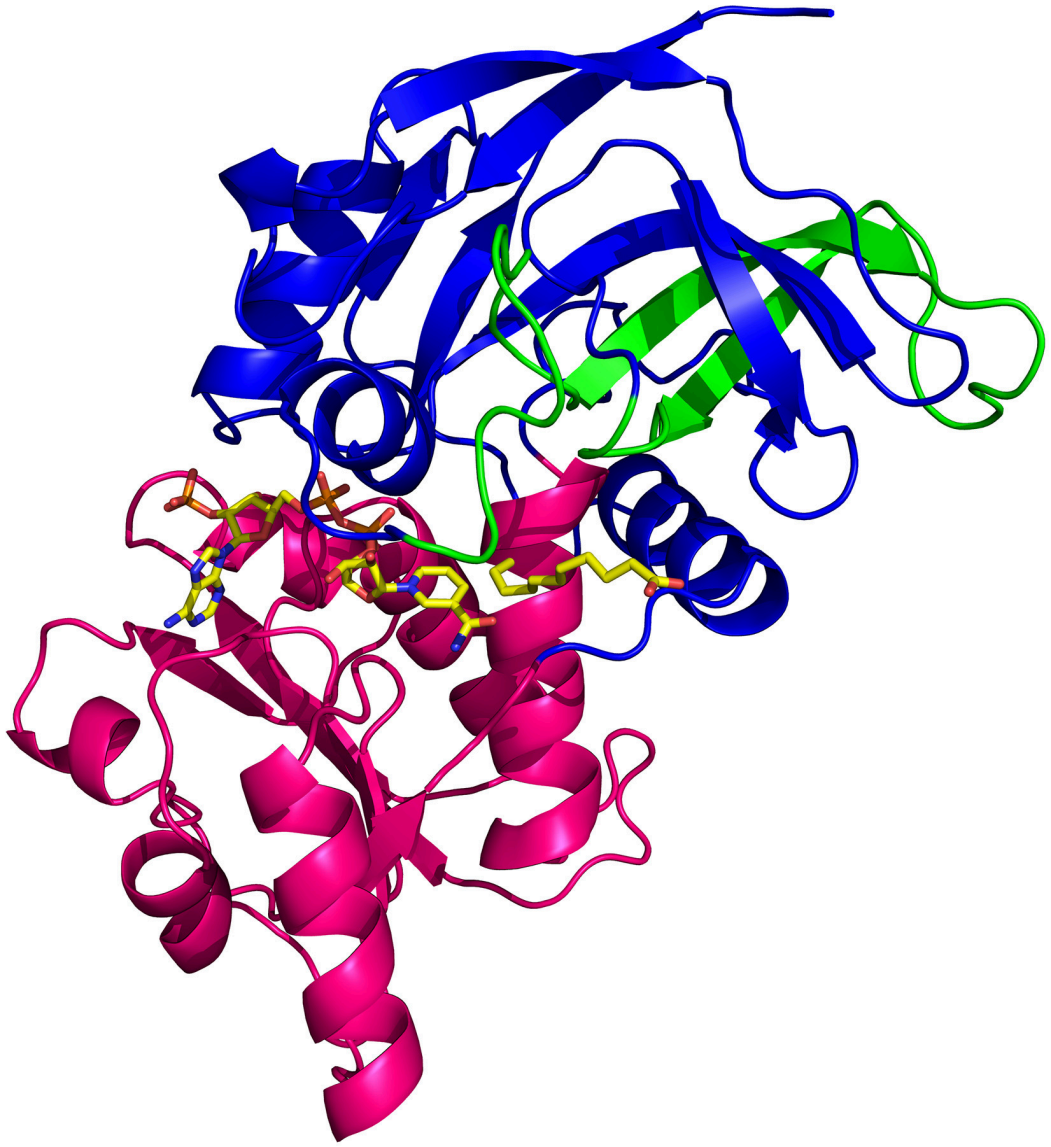
**View of the ceQORH monomer from ceQORH-NADP^+^-13-KOTE**. The catalytic domain (Gly3-Pro132, Leu277-Pro329) is colored in blue. The cofactor binding domain (Val133-Leu276) is colored in magenta. The internal chloroplast targeting sequence peptide (Pro59-Leu100) is colored in green. NADP^+^ and 13-KOTE are drawn in sticks.

The ceQORH monomer is formed of two domains (Figure 5) with (i) the catalytic domain including residues from Gly3 to Pro132 and from Leu277 to Pro329 and (ii) the cofactor binding domain (Val133 to Leu276) showing the classical motif of Rossmann fold. No major overall conformational change is observed in monomers following ligand binding, as shown by the low values of rmsd between monomers. Minor local changes could be observed following ligands binding, as described below.

#### Description of the ligand binding sites

**Figure 6:**
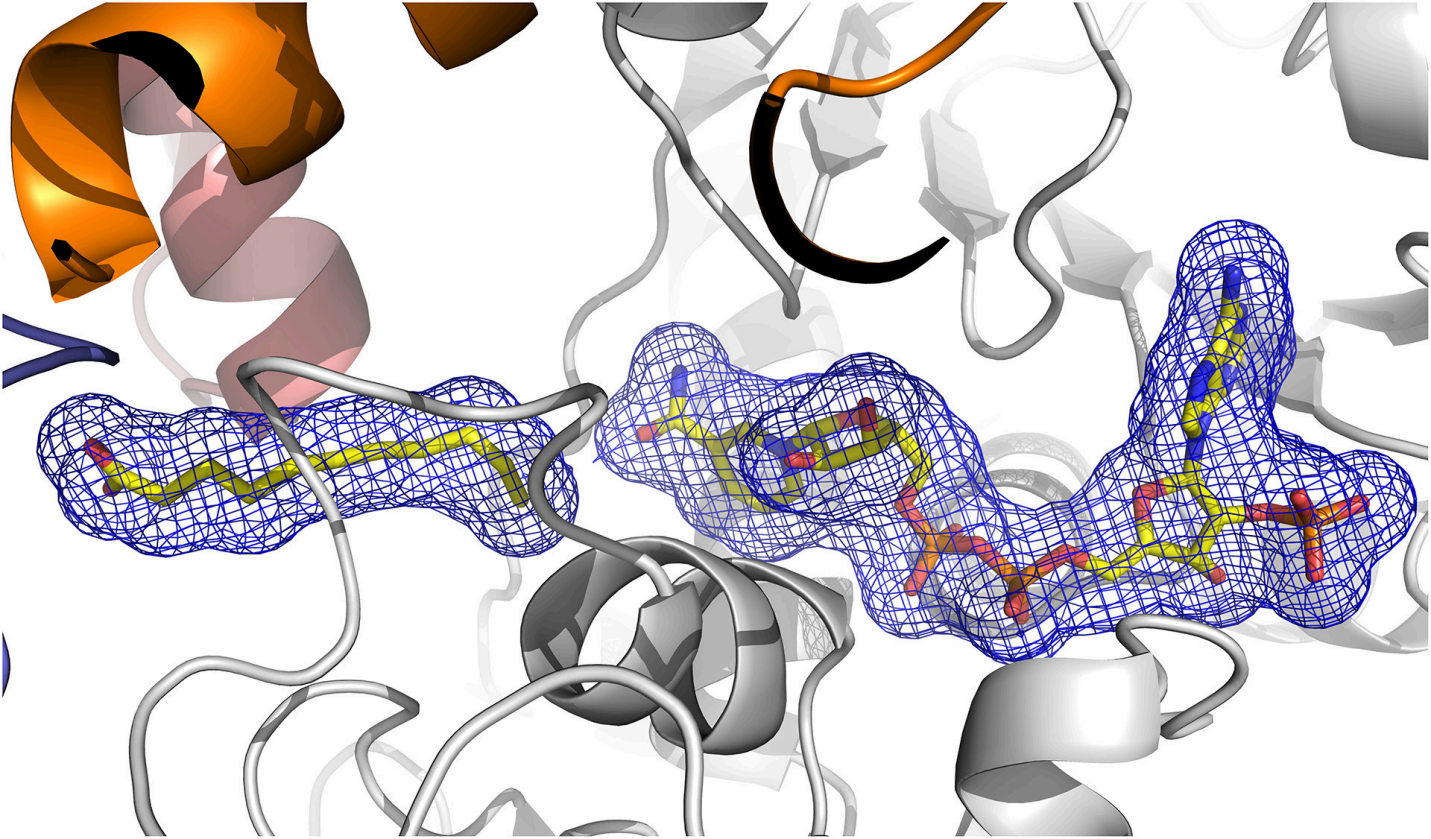
**View of the 2Fo-Fc electron density omit map (blue) at 2.8 Å resolution contoured at 1.2 σ level, calculated using PHENIX, surrounding 13-KOTE and NADP^+^**. The β-strands are drawn in arrows and the α-helices are represented in ribbons. 13-KOTE until the carbon C11 and NADP^+^ are drawn in stick.

Figure 6 shows that NADP^+^ is well-defined in the electron density map of the ternary complex (ceQORH-NADP^+^-13-KOTE). NADP^+^ is bound at the interface of the two domains and the nicotinamide ring is in the vicinity of the catalytic site. Comparison of apo-ceQORH and ceQORH-NADP^+^-13-KOTE monomers shows that NADP^+^ binding introduces local changes in the vicinity of the cofactor. The orientation of the Arg190 side chain is modified, stabilizing the 2′-phosphate of the NADP^+^ adenosine moiety. The loop containing Thr251 in the Rossmann fold shifts and stabilizes the ribose group of the NADP^+^ nicotinamide moiety by interacting with the Thr251 hydroxyl group.

**Figure 7:**
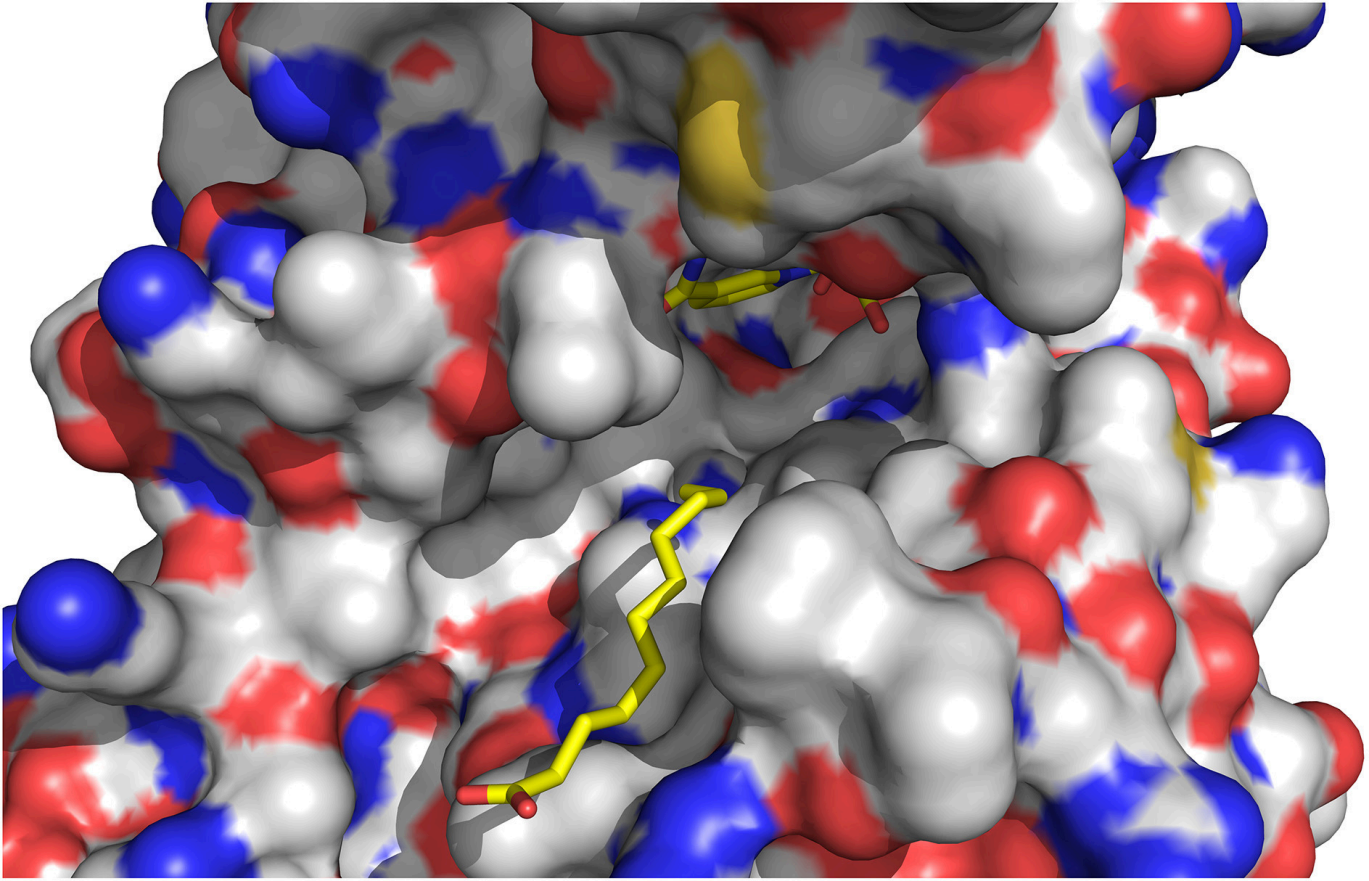
**View of the ligand binding site of ceQORH. 13-KOTE until the carbon C11 and NADP^+^ are drawn in stick**.

The quality of the electron density map for 13-KOTE differs depending on the monomers in the binary and ternary complexes, suggesting flexibility of the 13-KOTE aliphatic chain (Figure 6). The 13-KOTE binding site is large and solvent accessible (Figure 7). Upon 13-KOTE binding, the loop from Leu97 to Gly103 moves, thus stabilizing the ligand (Figure S3).

13-KOTE is not a substrate of ceQORH (Curien et al., 2016) though it displays an unsaturated double bond in α,β of a carbonyl group. Analyses of the ceQORH structures provide a possible explanation for this absence of activity. In the ceQORH-NADP^+^-13-KOTE structure, the aliphatic chain of 13-KOTE is observed in electron density up to the C11 atom of the α,β-unsaturated carbon-carbon bond (C11 = C12) (Figure 6). The distance average between the C11 atom and the C4 atom of the NADP^+^ nicotinamide ring bearing the hydride is 6.84 Å. The flexibility of 13-KOTE and the large distance between the α,β-unsaturated carbon-carbon bond (C11 = C12) and the C4 atom of NADP^+^ nicotinamide ring are not compatible with a hydride transfer.

#### Changes in the ceQORH oligomerization states

We previously showed (Mas y mas et al., 2015) that addition of NADPH to apo-ceQORH changes the oligomerization state of ceQORH from a mixture of monomers, dimers and tetramers to a solution of monomers. NADPH is always present *in vivo* and the dimeric apo-ceQORH probably never forms in cell. In the apo-ceQORH dimer, interactions between monomers mainly involve residues in the α-helix Pro254-Met267 belonging to the cofactor binding domain. Each α-helix in the dimer contributes for 723 Å^2^ to the buried area, being 60% of the overall buried area at the dimer interface. When the apo-ceQORH structure is compared either to the ceQORH-13-KOTE or ceQORH-NADP^+^-13-KOTE structure, the main chain from Asn46 to Leu61 and from Ile250 to Lys269, containing the α-helix Pro254-Met267 involved in the dimer interface, are shifted. The shift of the main chain from Ile250 to Lys269 is even larger in the ceQORH-NADP^+^-13-KOTE structure compared to the ceQORH-13-KOTE complex due to the NADP^+^ binding. The new position of the α-helix Pro254-Met267 observed in ceQORH-NADP^+^-13-KOTE could prevent the dimer formation when ceQORH binds NADPH (Figure S3) and may explain why ceQORH-NADPH behaves as a monomer in solution (Mas y mas et al., 2015).

**Figure 8:**
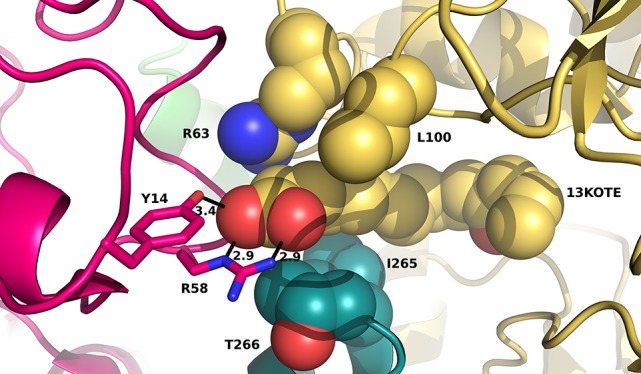
**View of residues stabilizing the first atoms of 13-KOTE in the ceQORH-13-KOTE**. 13-KOTE tetramer, Arg63 and Leu100 from one monomer, and Ile265, Thr266 from a second monomer are represented in van der Waals spheres. Arg58 and Tyr14 from a third monomer are drawn in stick. Hydrogen bonds between the 13-KOTE carboxylate group and Tyr14 and Arg58 are drawn in dashed lines and distances are given in Å.

As our results suggest that the tetrameric forms of ceQORH in the presence of 13-KOTE or 13-KOTE with NADP^+^ are non-physiological (see first paragraph), we briefly describe the structures. In the complexes ceQORH-13-KOTE and ceQORH-NADP^+^-13-KOTE complexes, residues of three ceQORH monomers interact with 13-KOTE by hydrogen bonds and van der Waals interactions. The 13-KOTE carboxylate group from one monomer is hydrogen bonded to the Arg58 guanidinium group and Tyr14 hydroxyl group from another monomer (Figure 8). Thus, the 13-KOTE carboxylate group is an anchoring point allowing for oligomerization in the crystals.

### Comparisons with other quinone oxidoreductases (QORs)

The apo-ceQORH monomer was used for searching homolog and comparisons with structures from the PDB using PDBefold (Krissinel and Henrick, 2004). When monomers are superimposed the values of rmsd go from 1.42 Å (comparison with the QOR from *C. burnetii*, PDB entry: 3TQH) (Franklin et al., 2015) to 2.65 Å (comparison with a putative NADPH quinone reductase from *Mesorhizobium loti*, PDB entry: 3PI7). The sequence identity based on structure comparison goes from 20 to 32%. The fold of QORs is roughly conserved and also similar to that of alcohol dehydrogenases belonging to the MDR family.

#### Comparison of oligomeric state

**Figure 9:**
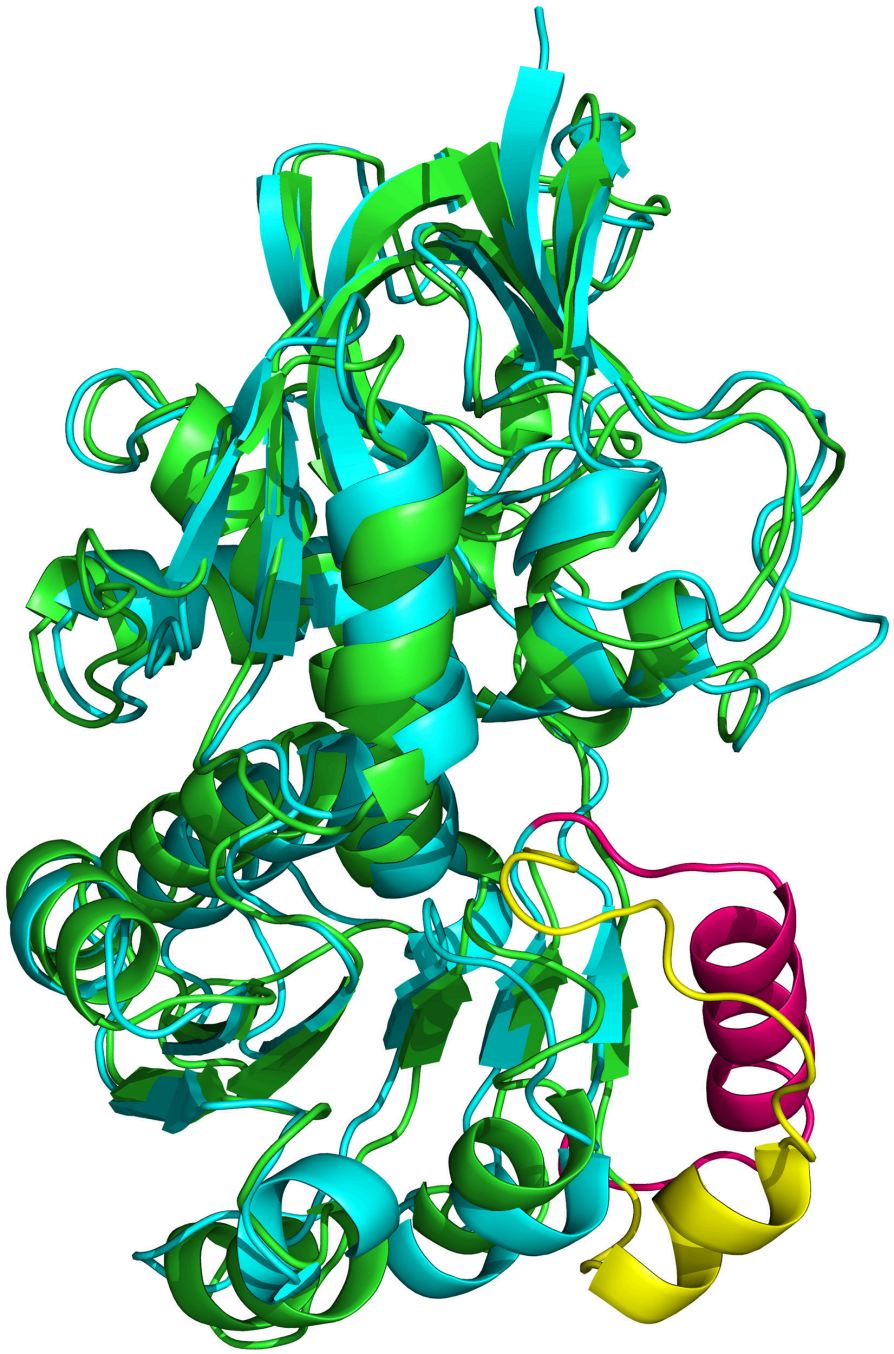
**Superimposition of the monomer from *E. coli* 1QOR (green) (Thorn et al., 1995) onto that of *A. thaliana* ceQORH (cyan)**. Gly239 to Ser258 in 1QOR is colored in yellow and the corresponding zone (Thr251-Lys269) in ceQORH is colored in pink.

Proteins in the QOR family display two different oligomeric states, being either monomers (such as the QOR from *C. burnetii)* or dimers [*E. coli* QOR (Thorn et al., 1995), *A. thaliana* alkenal reductase AtAER (Youn et al., 2006), *Saccharomyces cerevisiae* ζ-crystallin (Zta1) (Guo et al., 2011)]. Structure analysis provides an explanation for these differences. The main difference is observed when dimeric QORs are compared with apo-ceQORH. The analysis of dimeric QORs of known structures showed that interactions between the monomers occur by involving a β-strand of the Rossmann fold leading to the formation of a QOR dimer displaying a 12 stranded β-sheet. In ceQORH, the residues (Thr251-Lys269) are folded in a loop-α-helix(Pro254-Met267)-loop. This α-helix interacts with the non-crystallographic symmetry related α-helix in the apo-ceQORH dimer, as described above. Thr251-Lys269 from ceQORH do not superimpose onto the corresponding residues (Gly239 to Ser258) of QOR from *E. coli* (PDB entry: 1QOR) (Thorn et al., 1995) (Figure 9). The orientation of the α-helix (Pro254-Met267) prevents the monomer of ceQORH to interact with another monomer to form a dimer similar to that observed in dimeric QORs. When bound to NADP^+^ or NADPH, dimeric QORs remain in the dimeric state while AUC studies (Mas y mas et al., 2015) and tryptophan fluorescence anisotropy measurements showed that ceQORH is a monomer. Thus, dimeric QORs are stable dimers, which is not the case of ceQORH.

#### Comparison of the binding sites

When the cofactor binding site of other QORs is compared with that of ceQORH several interactions between these proteins and NADP^+^ are observed in the ceQORH-NADP^+^-13-KOTE structure. However, the residues in the catalytic site are not conserved at the exception of Asn46 which was proposed for substrate stabilization in *S. cerevisiae* ζ-crystallin (Zta1) (Guo et al., 2011). Ile50, Tyr59, and Leu131 proposed for quinone orientation in catalytic site of Zta1 correspond to the hydrophobic amino acids Val47, Ile56, and Val132 in ceQORH.

#### Insights into the discrimination of substrate length of the alkenal/alkenone reductases

**Figure 10:**
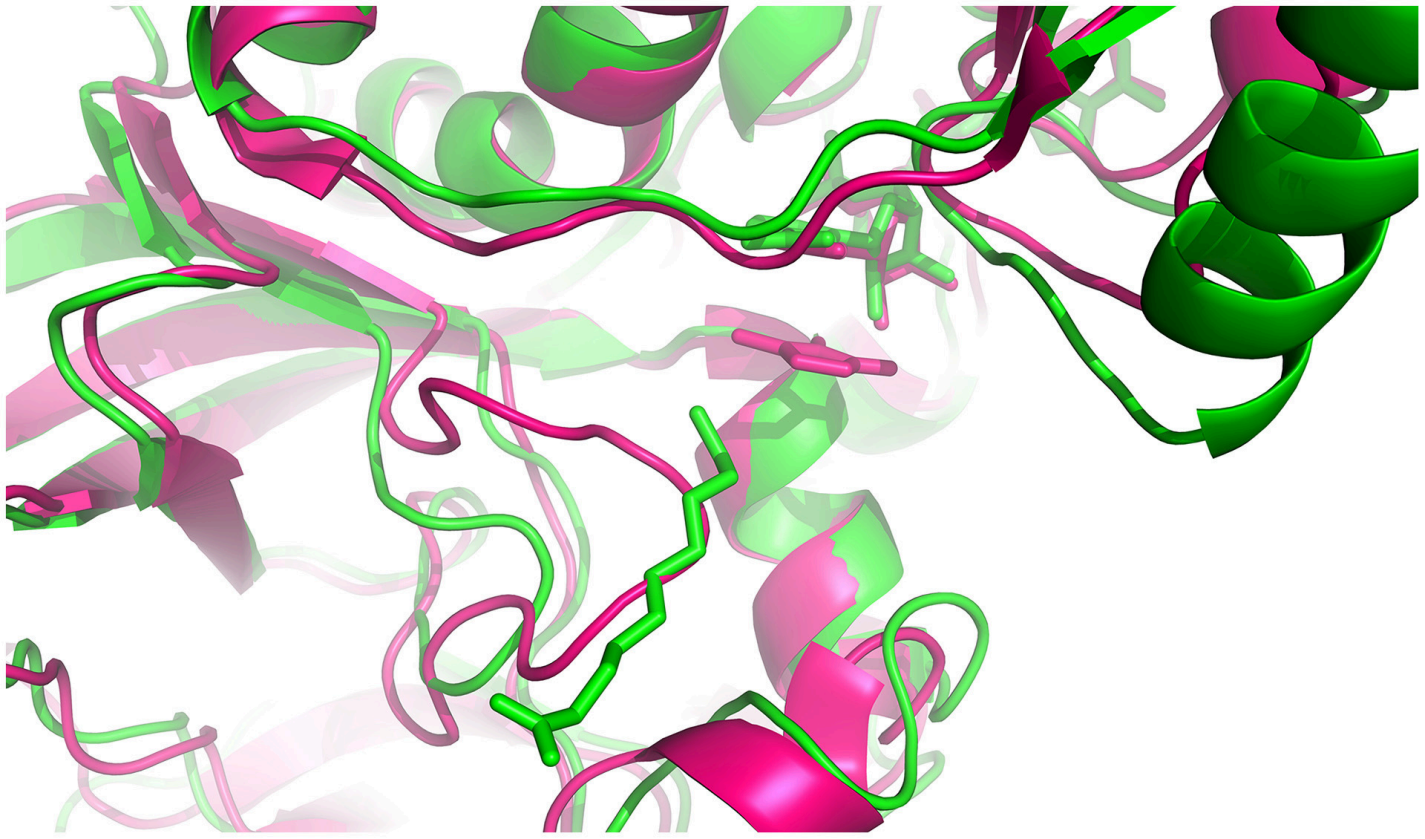
**Superimposition of the monomer from ceQORH-NADP^+^-13-KOTE (green) onto the enone reductase from *Fragaria x ananassa* (pink) (Schiefner et al., 2013)**. The cofactors and ligands are drawn in sticks. 4-hydroxy-5-methylfuran-3(2H)-one and NADPH from enone oxidoreductase are colored in pink. NADP^+^ and 13-KOTE from ceQORH are colored in green.

To gain a deeper understanding of the ligand specificity at the molecular level, structures of the *Arabidopsis* alkenal double bond reductase At5g16970 (AtAER) bound to *p*-coumaryl aldehyde or 4-hydroxy-2-nonenal (PDB entries: 2J3J and 2J3K, respectively) (Youn et al., 2006), of the enone oxidoreductase from *Fragaria x ananassa* (PDB entry: 4IDF) (Schiefner et al., 2013) and of ceQORH were compared. The enone oxidoreductase from *Fragaria x ananassa* (PDB entry: 4IDF) (Schiefner et al., 2013) displays 70.8% sequence identity with AtAOR (Figure S4). ceQORH shares 33% sequence identity with AtAOR and 32% with the enone oxidoreductase from *Fragaria x ananassa*. The rmsd values between the monomers of ceQORH-NADP^+^-13KOTE and the enone oxidoreductase from *Fragaria x ananassa* (PDB code: 4IDF) are between 1.48 and 1.52 Å. Analysis of the structures shows that the enzymes have a large substrate binding site. In 4IDF, the loop Leu104-Glu120 (Val112-Glu128 in AtAOR), corresponding to Leu97-Glu107 in ceQORH or Ile103-Glu108 in AtAER, is located in the vicinity of the binding site and is larger than in ceQORH and AtAER. In the six structures of enone oxidoreductase from *Fragaria x ananassa*, the conformation of this loop (B average of 13.3 Å^2^) does not change upon ligand binding and the loop protrudes inside the ligand binding site. Superimposition of the enone oxidoreductase from *Fragaria x ananassa* with the structures of AtAER and ceQORH shows clashes between the loop Leu104-Glu120 (Val112-Glu128 in AtAOR) and 13-KOTE of ceQORH-NADP^+^-13-KOTE (Figure 10), or with the *p*-coumaryl aldehyde in 2J3J and 4-hydroxy-2-nonenal in 2J3K of AtAER. The substrate specificity of AtAOR, restricted to small chain α,β-unsaturated carbonyl (C < 5), could therefore result from the length of the loop (Val112-Glu128) which could prevent binding of large substrates.

### ceQORH import sequence

ceQORH has the peculiar property to possess an internal signal sequence (Pro59-Leu100), for its import into the chloroplast, which is not cleaved after import (Miras et al., 2002, 2007). In the ceQORH structure, the internal chloroplast targeting sequence is folded as a two anti-parallel β-strands (Thr71 to Gly81 and Asp91 to Leu97) connected by a long loop (Ser82 to Gly90) (Figure 5). The chloroplast targeting sequence is partially solvent exposed and forms part of a larger 4-stranded β-sheet with the catalytic domain. Clearly, cleavage of this sequence would affect the active site and obviously had to be retained for the protein to be active. The internal chloroplast targeting sequence described for ceQORH has a similar fold in the other QORs. Therefore, the information for the ceQORH targeting likely does not rely on the structure of its internal chloroplast targeting sequence but rather on its primary structure.

## Discussion

9,12 γ-ketols are plant specific reactive electrophile oxylipins produced spontaneously in chloroplasts (Grechkin et al., 1991). Until recently, no enzyme able to detoxify γ-ketol in the chloroplast could be identified. Previously, we showed that ceQORH, a chloroplast inner envelope membrane associated protein, efficiently reduces γ-ketol but is inactive with the ketodienes 13-KODE and 13-KOTE and small reactive aldehydes (less than 9 carbon atoms) (Curien et al., 2016). Structure analyses of ceQORH crystallized bound to ligands showed that the ligand binding site is large and solvent exposed. These features are consistent with the binding of medium-sized (C > 9) molecules such as 1,3-diphenyl-2-propenone or 4-oxononenal but also with the binding of the long-chain molecules 9,12 γ-ketols (18 carbon atoms) (Curien et al., 2016). The structure analysis of the ternary complex ceQORH-NADP^+^-13-KOTE provides a structural explanation for the absence of activity with 13-KOTE with a positioning inconsistent with a hydride transfer from NADPH to the C = C bond positioned in α,β of the ketone. This observation also suggests that positioning of γ-ketol in the ligand binding site of ceQORH is different from that observed for 13-KOTE, γ-ketol being necessarily closer from the C4 of NADPH to be reduced. Structure comparisons and kinetic studies revealed that ceQORH shares structural similarities and overlapping substrate specificity with the cytoplasmic NADPH-dependent 2-alkenal reductase (AtAER) from *A. thaliana* (PDB entries: 2J3J and 2J3K) (Mano et al., 2005; Youn et al., 2006). AtAER is able to reduce the same substrates as ceQORH with similar efficiencies. However, AtAER substrate specificity is much larger than that of ceQORH, being active on ketodiene and ketotriene and showing much higher affinity for 4-hydroxy-2-nonenal and the C12 molecule traumatin (Curien et al., 2016). The structure comparison shows that both enzymes have a large ligand binding site. More generally, structure comparison with QORs and sequence alignments showed that the residues of ceQORH binding site are not well-conserved. Only co-crystallization of ceQORH and other QORs with substrates would allow understanding the basis of ceQORH restricted substrate specificity. Unfortunately we could not obtain crystals in the presence of γ-ketols. Nonetheless, by comparing different structures of QORs we could highlight a molecular basis allowing selection of the substrate length in the soluble stromal AtAOR, an enzyme that only reduces short-chain α,β unsaturated carbonyls. The AtAOR specificity for small compounds probably results from the presence of a long loop located in the vicinity of the binding site of AtAOR, which could prevent the binding of large molecules.

By comparison with other QORs that are stable dimers, all our results indicate that ceQORH is a monomer in solution and is active as a monomer. Oligomerization observed in crystals and in AUC experiments only occurs at high protein concentrations under conditions that are probably irrelevant of physiological conditions. Comparison of ceQORH structure with other dimeric QORs shows that the ceQORH dimer is different from the stable dimer of QORs due to structural differences in the Rossmann fold. A dimerization mode similar to that of AtAER is prevented in ceQORH by the orientation of the α-helix Pro254-Met267. Analysis of QOR from *C. Burnetii* (PDB entry: 3TQH) (Franklin et al., 2015) bound to NADPH, which is also a monomeric QOR supports this explanation as an α-helix equivalent to the ceQORH Pro254-Met267 α-helix is also observed. As ceQORH is associated to the inner membrane of chloroplast envelope (Miras et al., 2007), a monomeric state could increase its catalytic efficiency (higher mobility, better dispersion on the membrane surface) and might have been selected during evolution as this would favor the detoxification rate.

The successful crystallization of ceQORH in the presence of ketotriene and ketodiene raised the question of the physiological significance of this property. Kinetic experiments showed that ceQORH could be inhibited by 13-KODE. However, the inhibition was a slow process taking 400 s to reach completion A IC_50_ of 36 μM for 13-KODE could be determined in the presence of 25 μM substrate (Figures S5). 13-KODE and 13-KOTE are molecules that are produced during the hypersensitive response (HR) of plants attacked by a pathogen (Andersson et al., 2006). This response leads to local destruction of the plant tissues, preventing the spreading of the pathogen. Ketodiene and ketotriene can accumulate to 2.5–5 nmol per g fresh weight several hours after the hypersensitive response is initiated (Andersson et al., 2006). This value corresponds to 2.5–5 μM assuming a homogeneous distribution in the cell. As these compounds could be concentrated locally, they could reached concentration close to ceQORH IC_50_ and a first hypothesis might be that ceQORH sequesters ketodi(tri)enes quantitatively. However, this does not seem to be the case for the following reason: ceQORH is a low abundant protein at the cellular level. ceQORH represents about 1% of the chloroplast envelope proteins corresponding to about 50,000 copies per chloroplast, i.e., ~1 μM assuming the protein is soluble. This is barely compatible with an efficient sequestering of sub-micromolar concentrations of ketodiene and ketotriene. Another model can be proposed. Some RES oxylipins are signaling molecules (Farmer and Mueller, 2013). Upon increase of the ketodiene and ketotriene levels, following the triggering of the HR (Andersson et al., 2006), ceQORH would become inactivated by these molecules and would then be unable to metabolize γ-ketols. γ-ketols would in turn accumulate and contribute either to signaling or to an additional chemical defense of the plant. ceQORH may thus have a dual function, detoxification of γ-ketols under normal conditions and indirect contribution to chemical defense/signaling after pathogen attack. This would be reminiscent of the “floodgate” hypothesis proposed for H_2_O_2_ signaling where inhibition of 2-Cys peroxiredoxins occurs at high concentration of H_2_O_2_ (Wood et al., 2003).

More work is required to determine how ceQORH handles 13-KODE, 13-KOTE and γ-ketols *in vivo*, and whether ceQORH is involved during the HR response to pathogen attacks.

## Author contributions

SMM performed experiments, analyzed data and wrote the article. GC designed and performed experiments, analyzed data, and wrote the article. CG performed experiments. JLF and NR participated to the article writing. DC designed and performed experiments, analyzed data, and wrote the article. All authors read and approved the final manuscript.

### Conflict of interest statement

The authors declare that the research was conducted in the absence of any commercial or financial relationships that could be construed as a potential conflict of interest.

## Supplementary material

Figure S1 **Skeletal formulas of γ-ketol 18:1, 13-KOTE, 13-KODE and drawing of the reaction catalyzed by ceQORH**.

Figure S2 **Sequence alignment between ceQORH from *Arabidopsis thaliana*, and QORs from *Coxiella burnetii* (3TQH) (Franklin et al., 2015), *Escherichia coli* (1QOR) (Thorn et al., 1995), and *Saccharomyces cerevisiae* (3QWA) Guo et al., 2011**. The ceQORH secondary structure is drawn. The β-strands are represented by arrows and the α-helices by curls. The conserved residues are highlighted in red. The sequences were aligned using Multalin (Corpet, 1988) and the drawing was generated using ESPript (Gouet et al., 1999).

Figure S3 **Superimposition of apo-ceQORH (green), ceQORH-13-KOTE (cyan), and ceQORH-NADP^+^-13-KOTE (magenta) displaying conformational changes which occur upon binding of 13-KOTE and 13-KOTE + NADP^+^**.

Figure S4 **Sequence alignment between ceQORH, AtAER (Mano et al., 2002), AtAOR from *Arabidopsis thaliana* (Yamauchi et al., 2012), and the enone oxidoreductase from *Fragaria x ananassa* (4IDF) (Schiefner et al., 2013)**. The conserved residues are highlighted in red. The sequences were aligned using Multalin (Corpet, 1988) and the drawing was generated using ESPript (Gouet et al., 1999).

Figure S5 **Inhibition of ceQORH by the ketodiene 13-KODE; (A)** activity (ΔAbs340 nm/s) was measured after different preincubation time of the enzyme with 13-KODE. 50 nM enzyme was pre-incubated in the presence of 10 mM HEPES-KOH pH 7.5, 150 mM KCl, 160 μM NADPH and 55 μM 13-KODE. The reaction was initiated by the addition of 25 μM *trans*-1,3 diphenyl-2-propenone. The *k*obs value was obtained by nonlinear least-square fitting of the progress curves using the following equation: At = At0–vs.t + (vs–vi). (1-exp(-*k*obs.t))/*k*obs, where At is the absorbance at time t, At0 is the absorbance at t0, *v*i is the initial velocity of the reaction, vs. is the steady-state velocity of the reaction, and *k*obs is an exponential decay constant. A *k*obs value of 0,01 s-1 was obtained by curve fitting and t_1/2_ (i.e., ln2/*k*obs) was 70 s. **(B)** The enzyme (50 nM) was preincubated 400 s in the presence of 160 μM NADPH and 13-KODE at different concentrations as indicated in the graph before addition of the 25 μM *trans*-1,3 diphenyl-2-propenone. An IC50 of 36 μM (±3) μM was measured.

## Abbreviations

9-KODE: 9-oxo-10(E), 12(E)-octadecadienoic acid
13-KODE: 13-oxo-9(Z),11(E)-octadecadienoic acid
9-KOTE: 9-oxo-10(E), 12(Z), 15(Z)-octadecatrienoic acid
13-KOTE: 13-oxo-9(Z), 11(E), 15(Z)-octadecatrienoic acid
ceQORH: chloroplast envelope quinone oxidoreductase homolog
AtAER: alkenal/one reductase from *Arabidopsis thaliana* (cytosolic enzyme)
AtAOR: alkenone oxidoreductase from *Arabidopsis thaliana* (chloroplastic enzyme)
