# Supplementary figures and images for: Crystal Structure of the Chloroplastic Oxoene Reductase ceQORH from Arabidopsis thaliana
Source: Front Plant Sci. 2017 Mar 9;8:329. doi: 10.3389/fpls.2017.00329 (PMC5343027; doi:10.3389/fpls.2017.00329)

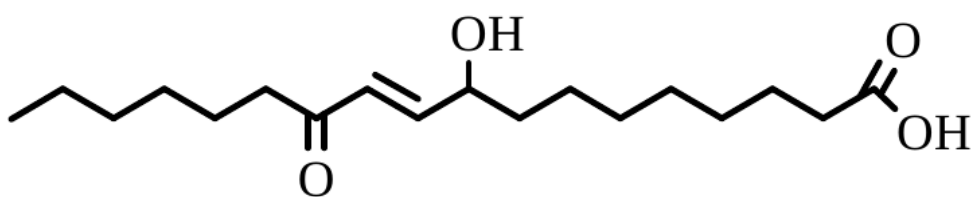

$\gamma$ -ketol 18:1

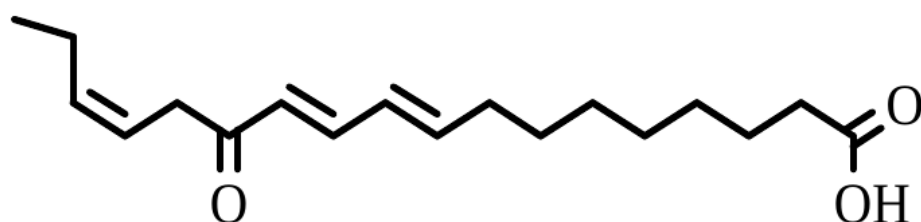

13KOTE

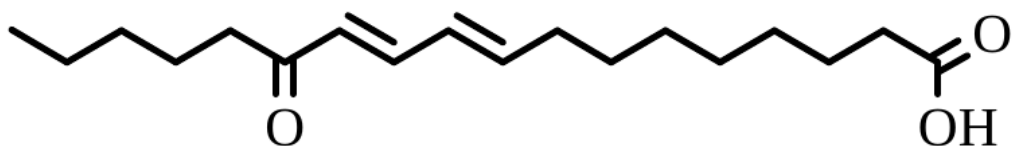

13KODE

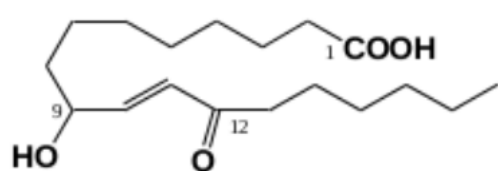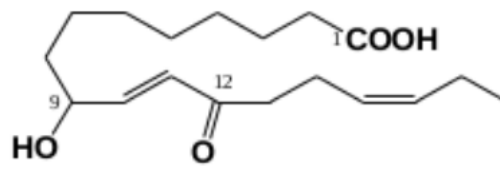

9,12  $\gamma$ -ketols

NADPH

NADP<sup>+</sup>

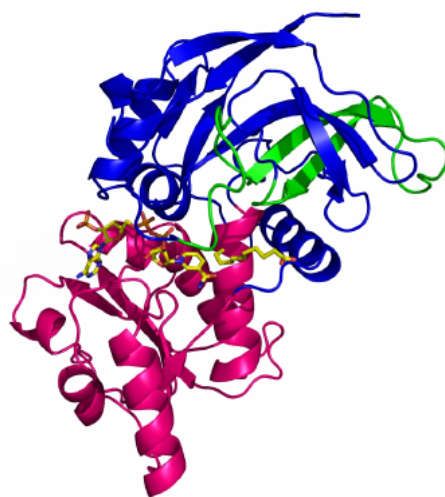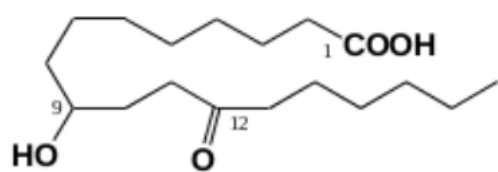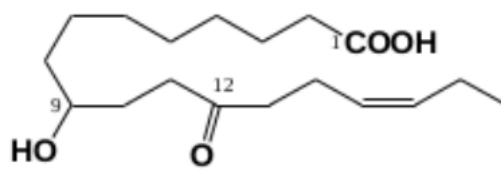

dihydro  $\gamma$ -ketols

Supplement: Figure S1 — Skeletal formulas of γ-ketol 18:1, 13-KOTE, 13-KODE and drawing of the reaction catalyzed by ceQORH. [file Image1.PDF]

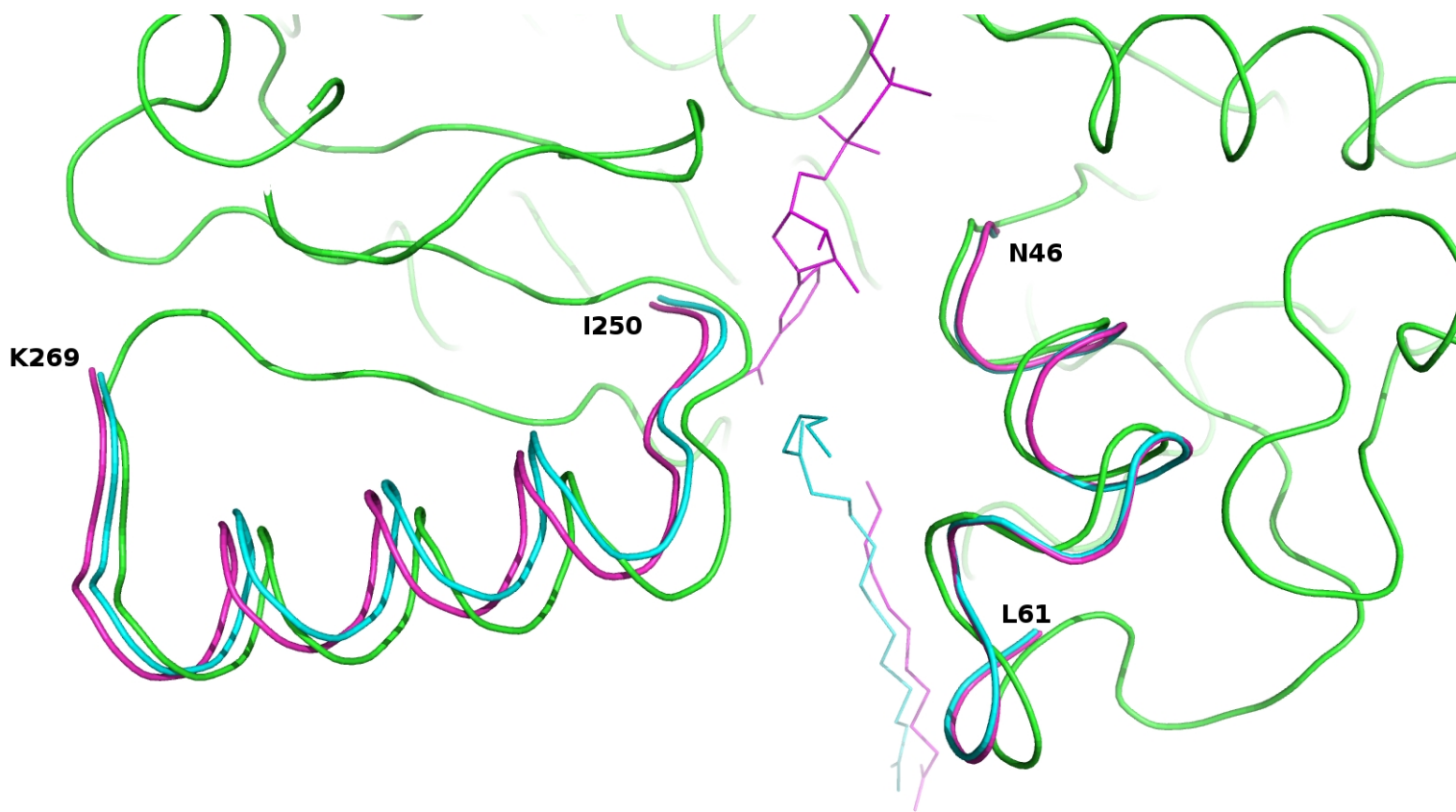

Supplement: Figure S3 — Superimposition of apo-ceQORH (green), ceQORH-13-KOTE (cyan), and ceQORH-NADP+-13-KOTE (magenta) displaying conformational changes which occur upon binding of 13-KOTE and 13-KOTE + NADP+. [file Image3.PDF]

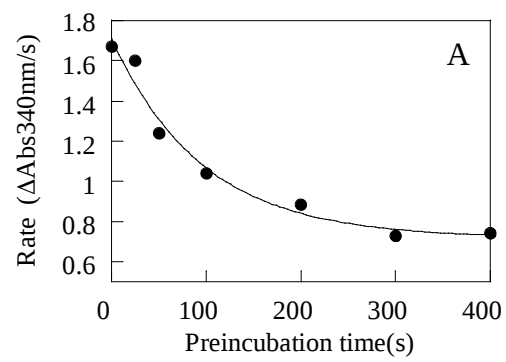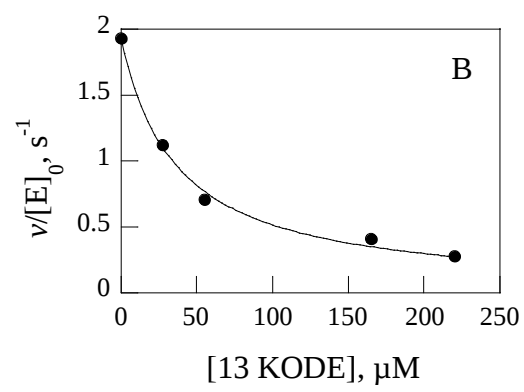

Supplement: Figure S5 — Inhibition of ceQORH by the ketodiene 13-KODE; (A) activity (ΔAbs340 nm/s) was measured after different preincubation time of the enzyme with 13-KODE. 50 nM enzyme was pre-incubated in the presence of 10 mM HEPES-KOH pH 7.5, 150 mM KCl, 160 μM NADPH and 55 μM 13-KODE. The reaction was initiated by the addition of 25 μM trans-1,3 diphenyl-2-propenone. The kobs value was obtained by nonlinear least-square fitting of the progress curves using the following equation: At = At0–vs.t + (vs–vi). (1-exp(-kobs.t))/kobs, where At is the absorbance at time t, At0 is the absorbance at t0, vi is the initial velocity of the reaction, vs. is the steady-state velocity of the reaction, and kobs is an exponential decay constant. A kobs value of 0,01 s-1 was obtained by curve fitting and t1/2 (i.e., ln2/kobs) was 70 s. (B) The enzyme (50 nM) was preincubated 400 s in the presence of 160 μM NADPH and 13-KODE at different concentrations as indicated in the graph before addition of the 25 μM trans-1,3 diphenyl-2-propenone. An IC50 of 36 μM (±3) μM was measured. [file Image5.PDF]
