# Supplementary material for: Crystal Structure of the Chloroplastic Oxoene Reductase ceQORH from Arabidopsis thaliana
Source: Front Plant Sci. 2017 Mar 9;8:329. doi: 10.3389/fpls.2017.00329 (PMC5343027; doi:10.3389/fpls.2017.00329)
Supplement: Figure S2 — Sequence alignment between ceQORH from Arabidopsis thaliana, and QORs from Coxiella burnetii (3TQH) (Franklin et al., 2015), Escherichia coli (1QOR) (Thorn et al., 1995), and Saccharomyces cerevisiae (3QWA) Guo et al., 2011. The ceQORH secondary structure is drawn. The β-strands are represented by arrows and the α-helices by curls. The conserved residues are highlighted in red. The sequences were aligned using Multalin (Corpet, 1988) and the drawing was generated using ESPript (Gouet et al., 1999). [file Image2.PDF]

```
ceQORH    β1  β2  β3  α1
          1  10  20  30  40  50
ceQORH    ...MAGKLMHALQYNSYGGGAAGLEHVQVPVPTPKSNEVCLKLEATSLNPVDWKIQKG..
3TQH      .....MKEMKAIQFDQFGPPKV.LKLVDTPTPEYRKNQMLIKVHAASLNPIDYKTRNGSG
1QOR      .......MATRIEFHKHGGPEV.LQAVEFTPADPAENEIQVENKAIGINFIDTYIRSG..
3QWA      MKCTIPEQQKVILIDEIGGYDV.IKYEDYPVPSISEEELLIKNKYTGVNYIESYFRKG..

ceQORH    β4  β5  β6
          60  70  80  90  100  110
ceQORH    MIRPFLPRKFPCIPATDVAGEVVEVGSGVKNFKAGDKVVAVLSHLGGGGL.AEFAVATEK
3TQH      FVAKKLKNNLPSGLGYDFSGEVIELGSDVNNVNIGDKVMGIAGFPDHPCCYAEYVCASPD
1QOR      ...LYPPPSLPSGLGTEAAGIVSKVGSGVKHIKAGDRVVY.AQSALGAYSSVHNIIADKA
3QWA      ...IYPCEK.PYVLGREASGTVVAKGKGVTNFEVGDQVAYISNSTFAQYSKISS..QGPV

ceQORH    β7  α2  α3  β8  α4
          120  130  140  150  160  170
ceQORH    LTVKRPQEVG.AAEAAALPVAGLTALQALTNPAGLKLDGTGKKANILVTAASGGVGHYAV
3TQH      TIIQKLEKLS.FLQAASLPTAGLTALQAL.NQAEVKQGDV.....VLIHAGAGGVGHLAI
1QOR      AILPAAIS...FEQAAASFLKGLTVYYLLRKTYEIKPDEQ.....FLFHAAAGGVGLIAC
3QWA      MKLPKGTSDEELKLYAAGLLQVLTALSFTNEAYHVKKGDY.....VLLFAAAGGVGLILN

ceQORH    β9  α5  β10  α6  β11
          180  190  200  210  220  230
ceQORH    QLAKLANAHVTATCG.ARNIEFVKSLGADEVLDYKTPEGAA..LKSPSGKKYDAVVHCAN
3TQH      QLAKQKGTTVITTAS.KRNHAFLKALGAEQCINYHEEDFLL..AISTP...VDAVIDLVG
1QOR      QWAKALGAKLIGTVGTAQKAQSALKAGAWQVINYREEDLVERLKEITGGKKVRVVYDSVG
3QWA      QLLKMKGAHTIAVASTDEKLKIAKEYGAEYLINASKEDILRQVLKFTNGKGVDASFDSVG

ceQORH    α7  β12  α8  β13
          240  250  260  270  280
ceQORH    GIPFSVFEPNLSENGKVIDITPGPNAMWTYAVKKITMS......KKQLVPLLLIPK..AE
3TQH      GDVGIQSIDCLKETGCIVSV...PTITAGRVIEVAKQK......HRRAFGLLKQFN..IE
1QOR      RDTWERSLDCLQRRGLMVSFGNSSGAVTGVNLGILNQKGSLYVTRPSLQGYITTREELTE
3QWA      KDTFEISLAALKRKGVFVSFGNASGLIPPFSITRLSPK.NITLVRPQLYGYIADPEEWKY

ceQORH    α9  β14  α10  β15
          290  300  310  320
ceQORH    NLEFMVNLVKEG..KVKTVIDSKHPLSKAEDAWAKSIDGHATGKIIVEP..
3TQH      ELHYLGKLVSED..KLRIEISRIFQLSEAVTAHELLETGHVRGKLVFKVR.
1QOR      ASNELFSLIASGVIKVDVAEQQKYPLKDAQRAHEILESRATQGSSLL.IP.
3QWA      YSDEFFGLVNSK..KLNIKIYKTYPLRDYRTAAADIESRKTVGKLVLEIPQ
```
