# Supplementary material for: Crystal Structure of the Chloroplastic Oxoene Reductase ceQORH from Arabidopsis thaliana
Source: Front Plant Sci. 2017 Mar 9;8:329. doi: 10.3389/fpls.2017.00329 (PMC5343027; doi:10.3389/fpls.2017.00329)
Supplement: Figure S4 — Sequence alignment between ceQORH, AtAER (Mano et al., 2002), AtAOR from Arabidopsis thaliana (Yamauchi et al., 2012), and the enone oxidoreductase from Fragaria x ananassa (4IDF) (Schiefner et al., 2013). The conserved residues are highlighted in red. The sequences were aligned using Multalin (Corpet, 1988) and the drawing was generated using ESPript (Gouet et al., 1999). [file Image4.PDF]

```
                                  1        10        20        30        40
ceQORH    ..................MAGKLMHALQYNSYGGGAAGLE.HVQVPVPTPKSNEVCLKLE
AtAER     .............MTATNKQVILKDYVSGFPTESDFDFTTTTVELRVPE.GTNSVLVKNL
4IDF      MASWSHPQFEKGAAAPSESIPSVNKAWVYSEYGKTSDVLKFDPSVAVPEIKEDQVLIKVV
AtAOR     .......................MKAWVYSDYGGV.DVLKLESNIVVPEIKEDQVLIKVV

                  50        60        70        80        90
ceQORH    ATSLNPVDWKIQKGMIRPFLPRKFPC.IPATDVAGEVVE..VGSGVKNFKAGDKVVAVLS
AtAER     YLSCDPY.MRIRMGKPDPSTAALAQAYTPGQPIQGYGVSRIIESGHPDYKKGDLLWGIVA
4IDF      AASLNPVDFKRALGYFKD.TDSPLPT.IPGYDVAGVVVK..VGSQVTKFKVGDEVYGDLN
AtAOR     AAALNPVDAKRRQGKFKA.TDSPLPT.VPGYDVAGVVVK..VGSAVKDLKEGDEVYANVS

         100       110       120       130       140       150
ceQORH    HLG......GGGLAEFAVATEKLTVKRPQEVGAAEAAALPVAGLTALQALTNPAGLKLDG
AtAER     WEE......YSVITPMTHAHFKIQHTDVPLSYYTGLLGMP..GMTAYAG.....FYEVCS
4IDF      ETALVNPTRFGSLAEYTAADERVLAHKPKNLSFIEAASLPLAIETAHE......GLERAE
AtAOR     EKALEGPKQFGSLAEYTAVEEKLLALKPKNIDFAQAAGLPLAIETADE......GLVRTE

                160       170          180       190         200
ceQORH    TGKKANILVTAASGGVGHYAVQLAK..LANAHVTATCGARNIEFVKS.LGADEVLDYKTP
AtAER     PKEGETVYVSAASGAVGQLVGQLAK.MMGCYVVGSAGSKEKVDLLKTKFGFDDAFNYKEE
4IDF      LSAGKSVLVLGGAGGVGTHIIQLAKHVFGASKVAATASTKKLDLLRT.LGADLAIDYTKE
AtAOR     FSAGKSILVLNGAGGVGSLVIQLAKHVYGASKVAATASTEKLELVRS.LGADLAIDYTKE

        210       220       230       240          250       260
ceQORH    E..GAALKSPSGKKYDAVVHCANGIPFSVFEPNLSENGKV.....IDITPGPNAMWTYAV
AtAER     SDLTAALKRCFPNGIDIYFENVGGKMLDAVLVNMNMHGRIAVCGMISQYNLENQEGVHNL
4IDF      N..FEDLPEKFDVVYDAVGETDKAVKA......VKEGGKV.....VTIVGPATP......
AtAOR     N..IEDLPDKYDVVFDAIGMCDKAVKV......IKEGGKV.....VALTGAVTP......

                270            280       290       300          310
ceQORH    KKITMSKKQLVPLLLIP....KAENLEFMVNLVKEGKVKTVIDSK..HPLSKAEDAWAKS
AtAER     SNIIYKRNRIQGFVVSDFYDKYSKFLEFVLPHIREGKITYVEDVA..DGLEKAPEALVGL
4IDF      ........PAILFVLTS....KGSVLEKLKPYLESGKVKPVLDPTSPYPFTKVVEAFGYL
AtAOR     ........PGFRFVVTS....NGDVLKKLNPYIESGKVKPVVDPKGPFPFSRVADAFSYL

           320
ceQORH    IDGHATGKIIVEP...
AtAER     FHGKNVGKQVVVVARE
4IDF      ESSRATGKVVVYPI..
AtAOR     ETNHATGKVVVYPIP.
```
